# Supplementary material for: Human plasma‐like medium enhances structural and metabolic maturation of human pluripotent stem cell‐derived cardiomyocytes
Source: Bioeng Transl Med. 2025 Nov 20;11(3):e70089. doi: 10.1002/btm2.70089 (PMC13247417; doi:10.1002/btm2.70089)
Supplement: Supplementary file 1 — Data S1. Supporting Information. [file BTM2-11-e70089-s003.docx]

**Supporting Information**

**Human Plasma-Like Medium Enhances Structural and Metabolic Maturation of Human Pluripotent Stem Cell-Derived Cardiomyocytes**

Xiaotian Zhang^1,3 *^, Aaron D. Simmons^2 *^, Kimberly S. Huggler^3,5^, Austin K. Feeney^1^, Vladislav Leonov^4^, Hee Jae Jang^4^, Timothy J Kamp^4^, Jason R. Cantor^1,3,5,6 #^, Melissa C. Skala^1,3 #^, Sean P. Palecek^2 #^

1. Department of Biomedical Engineering, University of Wisconsin-Madison, Madison, WI 53706, USA
2. Department of Chemical and Biological Engineering, University of Wisconsin-Madison, Madison, WI 53706, USA
3. Morgridge Institute for Research, Madison, WI 53715, USA
4. Department of Medicine, School of Medicine and Public Health, University of Wisconsin-Madison
5. Department of Biochemistry, University of Wisconsin-Madison, Madison, WI 53706, USA
6. Carbone Cancer Center, University of Wisconsin-Madison, Madison, WI 53705, USA

* These authors contributed equally to this work

^#^Correspondence:

sppalecek@wisc.edu, 3637 Engineering Hall, 1415 Engineering Drive, Madison, WI 53706

mcskala@wisc.edu, 3262 Morgridge Institute for Research, 330 N Orchard St, Madison, WI 53715

jcantor@morgridge.org, 2264 Morgridge Institute for Research, 330 N Orchard St, Madison, WI 53715

**Table S1. Reagents and resources used in this study**

| REAGENT or RESOURCE | SOURCE | IDENTIFIER |
| --- | --- | --- |
| **Antibodies** | | |
| rb-IgG-anti-MYH7 | R&D Systems | Cat#MAB90961100 |
| ms-IgG1-anti-MYH6 | R&D Systems | Cat#MAB8979 |
| ms-IgG1 anti-cTnT | ThermoFisher | Ca#MA5–12960 |
| Alexa Fluor 488-conjugated anti-mouse IgG1 | ThermoFisher | Cat# A-21121 |
| mouse IgG2b anti-MLC2A | Synaptic Systems | Cat #311011 |
| rabbit IgG anti-MLC2V | Proteintech | Cat#10906-1-AP |
| Alexa Fluor 488 anti-mouse IgG2b | ThermoFisher | Cat# A11008 |
| Alexa Fluor 647 anti-rabbit IgG | ThermoFisher | Cat# A21240 |
| mouse IgG1 anti-α-actinin | Sigma | Cat# A7811 |
| Alexa Fluor 488 anti-mouse IgG2b | ThermoFisher | Cat#A21141 |
| Alexa Fluor 647 anti-mouse IgG1 | ThermoFisher | Cat#A21240 |
| **Chemicals, peptides, and recombinant proteins** | | |
| Growth Factor Reduced Matrigel | Corning | Cat#354263 |
| Versene | Life Technologies | Cat#15040066 |
| Accutase | Innovative Cell Technology | Cat#AT104 |
| Y-27632 | Tocris | Cat#1254 |
| CHIR99021 | Selleckchem | Cat#S1263 |
| IWP2 | Tocris | Cat#3533 |
| Fetal bovine serum | R&D Systems | Cat#S12450 |
| DMSO | Sigma-Aldrich | Cat#D2650 |
| Paraformaldehyde | Electron Microscopy Sciences | Cat#15710-S |
| Bovine Serum Albumin | ThermoFisher | Cat#BP1600 |
| Triton X-100 | ThermoFisher | Cat#BP151 |
| Hoechst 33342 | Invitrogen | Cat#H3570 |
| Trizol Reagent | ThermoFisher | Cat#15596018 |
| RNaseOUT Recombinant Ribonuclease Inhibitor | Life Technologies | Cat#10777-019 |
| Oligo dT(20) primers | Life Technologies | Cat#18418020 |
| PowerUp SYBR Green Master Mix for qPCR | ThermoFisher | Cat#25780 |
| **Experimental models: Cell lines** | | |
| Human: IMR90-4 hiPSC line | N/A | N/A |
| Human: WTC11 hiPSC line | N/A | N/A |
| Human: H9 hESC line | N/A | N/A |
| **Software and algorithms** | | |
| MUSCLEMOTION (version 1.1) | N/A | https://github.com/l-sala/MUSCLEMOTION |
| Sotatool | N/A | https://github.com/steinjm/SotaTool |
| ImageJ | N/A | https://imagej.nih.gov/ij/ |
| GraphPad Prism (version 9.4.1) | N/A | https://graphpad.com |
| BioRender | N/A | https://biorender.com |
| Nikon Instruments Software Elements (version 5.30.06) | N/A | https://www.microscope.healthcare.nikon.com/products/software/nis-elements |
| MetaboAnalyst (version 6.0) | N/A | https://www.metaboanalyst.ca/MetaboAnalyst/ModuleView.xhtml |
| **Critical Commercial Assays** | | |
| Zymo RNA Clean & Concentrator-25 | Zymo Research | Cat#R1018 |
| Qiagen Omniscript RT Kit | Qiagen | Cat#205113 |
| Seahorse XF Cell Mito Stress Kit | Agilent Technologies | N/A |
| FLIPR® Calcium 6 Assay Kits | Molecular Devices | Ca#RB190 |
| Sequencing libraries prep | Takara | Ca#634411 |
| RNA sequencing | Illumina NovaSeq6000 | N/A |
| **Other** | | |
| mTeSR1 medium | STEMCELL Technologies | Cat#85850 |
| DMEM/F12 medium | ThermoFisher | Cat#11330032 |
| Standard RPMI1640 | Life Technologies | Cat#11875119 |
| Glucose free RPMI 1640 | Life Technologies | Cat#11879020 |
| HPLM | Rossiter et al. 2021^1^ | See Table S3 |
| B27 minus insulin supplement | Life Technologies | Cat#0050129SA |
| B27 plus insulin supplement | Life Technologies | Cat#17504044 |
| Mr. Frosty Freezing Container | ThermoFisher | Cat#51000001 |
| DPBS | ThermoFisher | Cat#14190144 |

**Table S2. Primer Sequences for qPCR**

| **Target** | **FW Primer (5’-3’)** | **RV Primer (5’-3’)** |
| --- | --- | --- |
| *ZNF384* | AATCTGCAGTCCCACAGACG | ACTGTGTGCGTAGACAGGTG |
| *EDF1* | CCAAGCAGGCTATCTTAGCGG | GACCTTGCTGGATCACCTTG |
| *DDB1* | TCAACGGCATGATAGGGCTG | CGCTCGGTGTGAAAGGATCT |
| *TNNT2* | TTCACCAAAGATCTGCTCCTCGCT | TTATTACTGGTGTGGAGTGGGTGTGG |
| *MYH6* | AGCTCACCTACCAGACAGAGG | TTGCTTGGCACCAATGTCAC |
| *MYH7* | GAGGAGCAAGCCAACACCAA | CTCATTCAAGCCCTTCGTGC |
| *MYL7* | GGTGGTGAACAAGGATGAGTT | GTGACTTGTAGTCGATGTTCCC |
| *MYL2* | GCTGAAGGCTGATTACGTTCG | TCCAAGTTGCCAGTCACGTC |
| *TNNI1* | GCAAACTCTTGCTGAAGAGCC | GTGTTGTGGAGGCATTTGGC |
| *TNNI3* | TGCAGATGCCATGATGCAGG | CCCGGTTTTCCTTCTCGGTG |
| *SYNPO* | GGGATCGAGGCTCAGGACC | GGCTCACCCAGCCGTCTA |
| *RRAD* | CGACTCAGACGAGAGCGTTT | GATCATAGGTGTGCCCTGCT |
| *GPX3* | ATGGGCAATCCCCAGATGGAC | GACCGAATGGTGCAAGCTCT |
| *PCDH20* | TGGGAAGCCACCCAGAGAAT | TTTCGTCCAGCGTCAGCATC |
| *CSRP3* | CCACACAGGCAGACTTGACC | CTGTCGTGCTGTCAAGAGCC |
| *CAV2* | GCTGTCTGCACATCTGGATTTTA | AATCCTGGCTCAGTTGCAGG |
| *MFN2* | GCTCGCTGGTGACGTAGTGA | TGTCTCAGGTTGAGGTTGGC |
| *PLN* | ATCACAGCTGCCAAGGCTAC | TGACGTGCTTGTTGAGGCAT |
| *GJA5* | AGCACATGGCTAAGTGCCAG | TCGTACTTGCTCGGTGACCA |
| *CASQ2* | GACGACTTTCCTCTGCTCGTT | CAGCTCCTCAGCAGTTGGAA |
| *SLC6A8* | CTGGGAGGTGACCCTTTGTCT | TAAATGATGCCATCCAGGGCG |
| *ITGA5* | AGACTTTCTTGCAGCGGGAG | ATCCACAGTGGGACGCCATA |
| *TNC* | GGACTCCTGTACCCCTTCCC | TGCGTCTCAGGAACACAATCC |
| *HIF1A* | GCAGAATGCTCAGAGAAAGCGA | GCTGCATGATCGTCTGGCTG |
| *MEF2C* | TGACTGTGAGATTGCGCTGA | CTTCTTTCTCAACGTCTCCACG |
| *MRPS6* | CACAACAGAGGCGGGTATTTCT | CGAGTGGGACTGGGACAATC |
| *S100A4* | CTGACTGCTGTCATGGCGTG | CAGCTTCATCTGTCCTTTTCCCC |
| *OPTN* | GAAAGGCCCGGAGACTGTTG | TCCTTTCAAGGGCCTGACAC |
| *ACADVL* | TTGTCCACCCGGAGTTGAGT | TGCAGCAGAAACTGTTCATTGAC |
| *DECR1* | TGGAAGCCATGAGCAAGTCTC | GGCGACCACAGGGAATTCTG |
| *PPARGC1A* | TGAACTGAGGGACAGTGATTTC | CCCAAGGGTAGCTCAGTTTATC |
| *nucDNA* | CAACTTCATCCACGTTCACC | GAAGAGCCAAGGACAGGTAC |
| *mitoDNA* | CGAAAGGACAAGAGAAATAAGG | CTGTAAAGTTTTAAGTTTTATGCG |

**Table S3. Basal HPLM formulation**

| **Concentrated stocks of components were prepared and pooled as described below** | | | | |
| --- | --- | --- | --- | --- |
| **All working concentrations in uM** | | | | |
| Components added relative to initial formulation described before^2^ | | | | |
|  | Concentration | Vendor | Product # | Stock solution pool (Storage) |
| ***Salts*** | | | | |
| CaCl2 | 2350 | Sigma-Aldrich | C5670 | 10x (-20°C) |
| KCl | 4100 | Sigma-Aldrich | P5405 | 10x (-20°C) |
| MgCl2 | 480 | Sigma-Aldrich | M8266 | 10x (-20°C) |
| MgSO4 | 350 | Sigma-Aldrich | M2643 | 10x (-20°C) |
| NaCl | 105000 | Sigma-Aldrich | S7653 | 10x (-20°C) |
| NaHCO3 | 24000 | Sigma-Aldrich | S5761 | 10x (-20°C) |
| Na2HPO4 | 870 | Sigma-Aldrich | S9390 | 10x (-20°C) |
| Ca(NO3)2 | 40 | Sigma-Aldrich | C1396 | 100x (-20°C) |
| NH4Cl | 40 | Sigma-Aldrich | A9434 | 100x (-20°C) |
| ***Metabolites*** | | | | |
| Histidine | 110 | Sigma-Aldrich | H5659 | 500x (-20°C) prepared in 0.1M HCl |
| Isoleucine | 70 | Sigma-Aldrich | I2752 | 500x (-20°C) prepared in 0.1M HCl |
| Leucine | 160 | Sigma-Aldrich | L8000 | 500x (-20°C) prepared in 0.1M HCl |
| Lysine | 200 | Sigma-Aldrich | L5626 | 500x (-20°C) prepared in 0.1M HCl |
| Methionine | 30 | Sigma-Aldrich | M9625 | 500x (-20°C) prepared in 0.1M HCl |
| Phenylalanine | 80 | Sigma-Aldrich | P2126 | 500x (-20°C) prepared in 0.1M HCl |
| Threonine | 140 | Sigma-Aldrich | T8625 | 500x (-20°C) prepared in 0.1M HCl |
| Tryptophan | 60 | Sigma-Aldrich | T0254 | 500x (-20°C) prepared in 0.1M HCl |
| Valine | 220 | Sigma-Aldrich | V0500 | 500x (-20°C) prepared in 0.1M HCl |
| Alanine | 430 | Sigma-Aldrich | A7627 | 500x (-20°C) |
| Arginine | 110 | Sigma-Aldrich | A5131 | 500x (-20°C) |
| Asparagine | 50 | Sigma-Aldrich | A0884 | 500x (-20°C) |
| Cysteine | 40 | Sigma-Aldrich | C1276 | 500x (-20°C) |
| Glycine | 300 | Sigma-Aldrich | G7126 | 500x (-20°C) |
| Proline | 200 | Sigma-Aldrich | P0380 | 500x (-20°C) |
| Serine | 150 | Sigma-Aldrich | S4500 | 500x (-20°C) |
| Aspartate | 20 | Sigma-Aldrich | A9256 | 500x (-20°C) prepared in 1M HCl |
| Cystine | 100 | Sigma-Aldrich | C8755 | 500x (-20°C) prepared in 1M HCl |
| Glutamate | 80 | Sigma-Aldrich | G1251 | 500x (-20°C) prepared in 1M HCl |
| Tyrosine | 80 | Sigma-Aldrich | T3754 | 500x (-20°C) prepared in 1M HCl |
| Glutamine | 550 | Sigma-Aldrich | G3126 | 250x (-20°C) |
| 4-hydroxyproline | 20 | Sigma-Aldrich | H5534 | 500x (-20°C) |
| Acetylcarnitine | 5 | Sigma-Aldrich | A6706 | 500x (-20°C) |
| Acetylglycine | 90 | Sigma-Aldrich | A16300 | 500x (-20°C) |
| alpha-Aminobutyrate | 20 | Sigma-Aldrich | A2536 | 500x (-20°C) |
| Betaine | 70 | Sigma-Aldrich | 61962 | 500x (-20°C) |
| Carnitine | 40 | Sigma-Aldrich | C0283 | 500x (-20°C) |
| Citrulline | 40 | Sigma-Aldrich | C7629 | 500x (-20°C) |
| Ornithine | 70 | Sigma-Aldrich | O2375 | 500x (-20°C) |
| Taurine | 90 | Sigma-Aldrich | T0625 | 500x (-20°C) |
| 2-hydroxybutyrate | 50 | Sigma-Aldrich | 220116 | 250x (-20°C) |
| 3-hydroxybutyrate | 50 | Sigma-Aldrich | 298360 | 250x (-20°C) |
| Acetate | 40 | Sigma-Aldrich | S5636 | 250x (-20°C) |
| alpha-Ketoglutarate | 5 | Sigma-Aldrich | 75892 | 250x (-20°C) |
| Citrate | 130 | Sigma-Aldrich | 251275 | 250x (-20°C) |
| Lactate | 1600 | Sigma-Aldrich | L7022 | 250x (-20°C) |
| Malate | 5 | Sigma-Aldrich | M7397 | 250x (-20°C) |
| Malonate | 10 | Sigma-Aldrich | M1296 | 250x (-20°C) |
| Pyruvate | 50 | Sigma-Aldrich | P2256 | 250x (-20°C) |
| Succinate | 20 | Sigma-Aldrich | S3674 | 250x (-20°C) |
| Creatine | 40 | Sigma-Aldrich | C0780 | 500x (-20°C) |
| Creatinine | 75 | Sigma-Aldrich | C4255 | 500x (-20°C) |
| Glutathione | 25 | Sigma-Aldrich | G6013 | 500x (-20°C) |
| Fructose | 40 | Sigma-Aldrich | F3510 | 500x (-20°C) |
| Galactose | 60 | Sigma-Aldrich | G5388 | 500x (-20°C) |
| Acetone | 60 | Sigma-Aldrich | AX0120 | 5000x (-20°C) |
| Formate | 50 | Sigma-Aldrich | 94318 | 5000x (-20°C) |
| Glycerol | 120 | Sigma-Aldrich | G2025 | 5000x (-20°C) |
| Hypoxanthine | 10 | Sigma-Aldrich | H9377 | 1000x (-20°C) prepared in 0.2M HCl |
| Uridine | 3 | Sigma-Aldrich | U3003 | 1000x (-20°C) prepared in 0.2M HCl |
| Uric acid | 350 | Sigma-Aldrich | U2625 | 250x Prepared fresh in 1M NaOH |
| Urea | 5000 | Sigma-Aldrich | U5378 | 250x Prepared fresh |
| Glucose | 5000 | Thermo Fisher | 15023-021 | 100x Prepared fresh |
| ***Vitamins*** | | | | |
| Indicated vitamin concentrations are according to supplementation with RPMI 1640 100X vitamin mix (Sigma-Aldrich-Aldrich R7256) | | | | |
| Concentrations significantly different versus expected values based on metabolite profiling, as described before^3^ | | | | |
| Biotin | 0.82 |  |  |  |
| Choline | 21.49 |  |  |  |
| Folic acid | 2.27 |  |  |  |
| Inositol | 194.27 |  |  |  |
| Niacinamide | 8.19 |  |  |  |
| p-Aminobenzoate | 7.29 |  |  |  |
| Pantothenate | 1.05 |  |  |  |
| Pyridoxine | 4.86 |  |  |  |
| Riboflavin | 0.53 |  |  |  |
| Thiamine | 2.96 |  |  |  |
| Vitamin B-12 | 0.004 |  |  |  |
| ***Other*** | | | | |
| Phenol red | 14 | Sigma-Aldrich | P5530 | 100x (-20°C) |

**Table S4. Summary statistics and test results for key assessments**

**Table S4 (continued). Summary statistics and test results for key assessments**

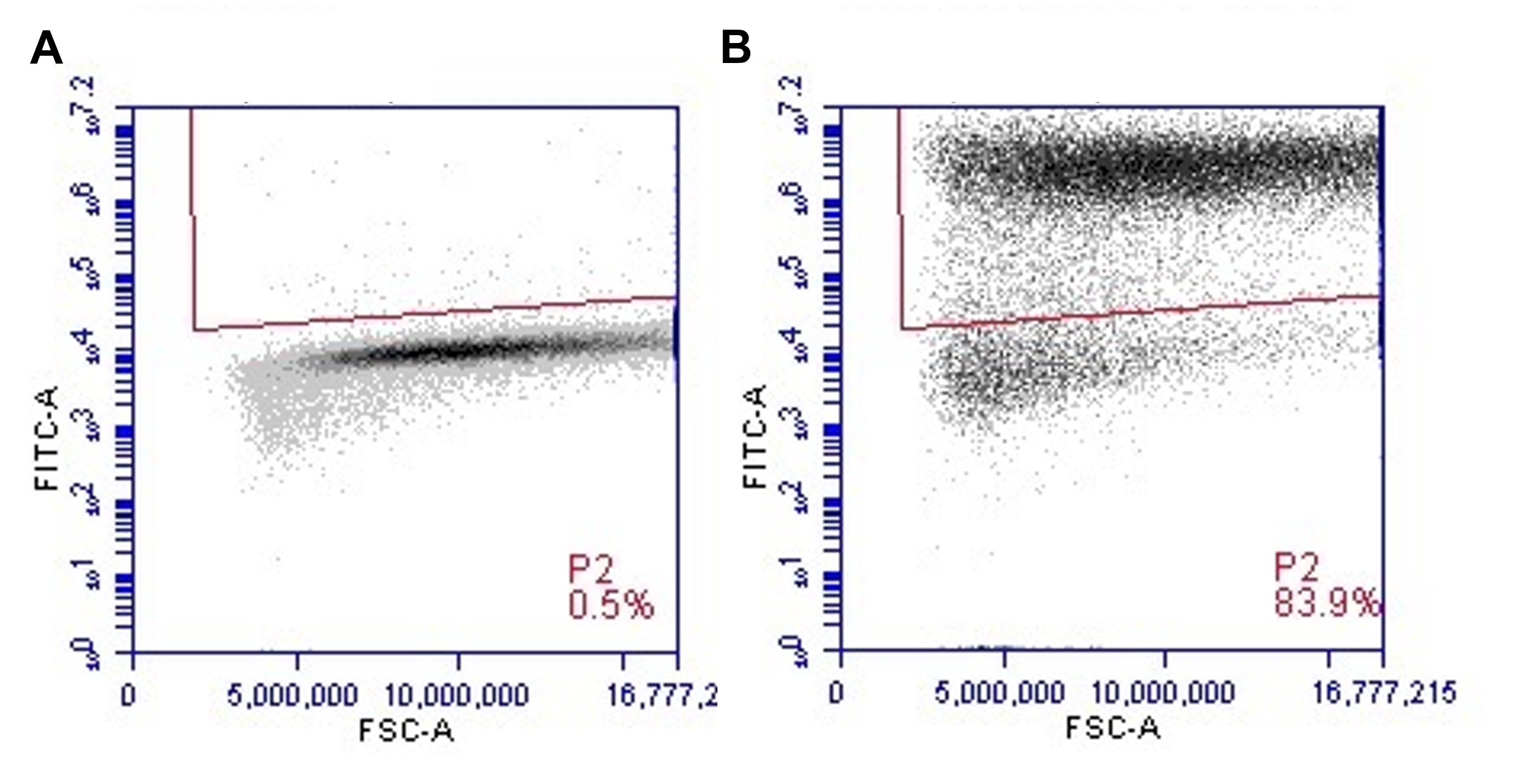


**Figure S1. Flow cytometry assessment of cardiomyocyte purity in Day 16 hPSC-CMs.** (A-B) Representative flow cytometry dot plots comparing (A) undifferentiated hPSCs (negative control) and (B) Day 16 hPSC-CMs stained for the cardiomyocyte-specific marker cTnT.

**
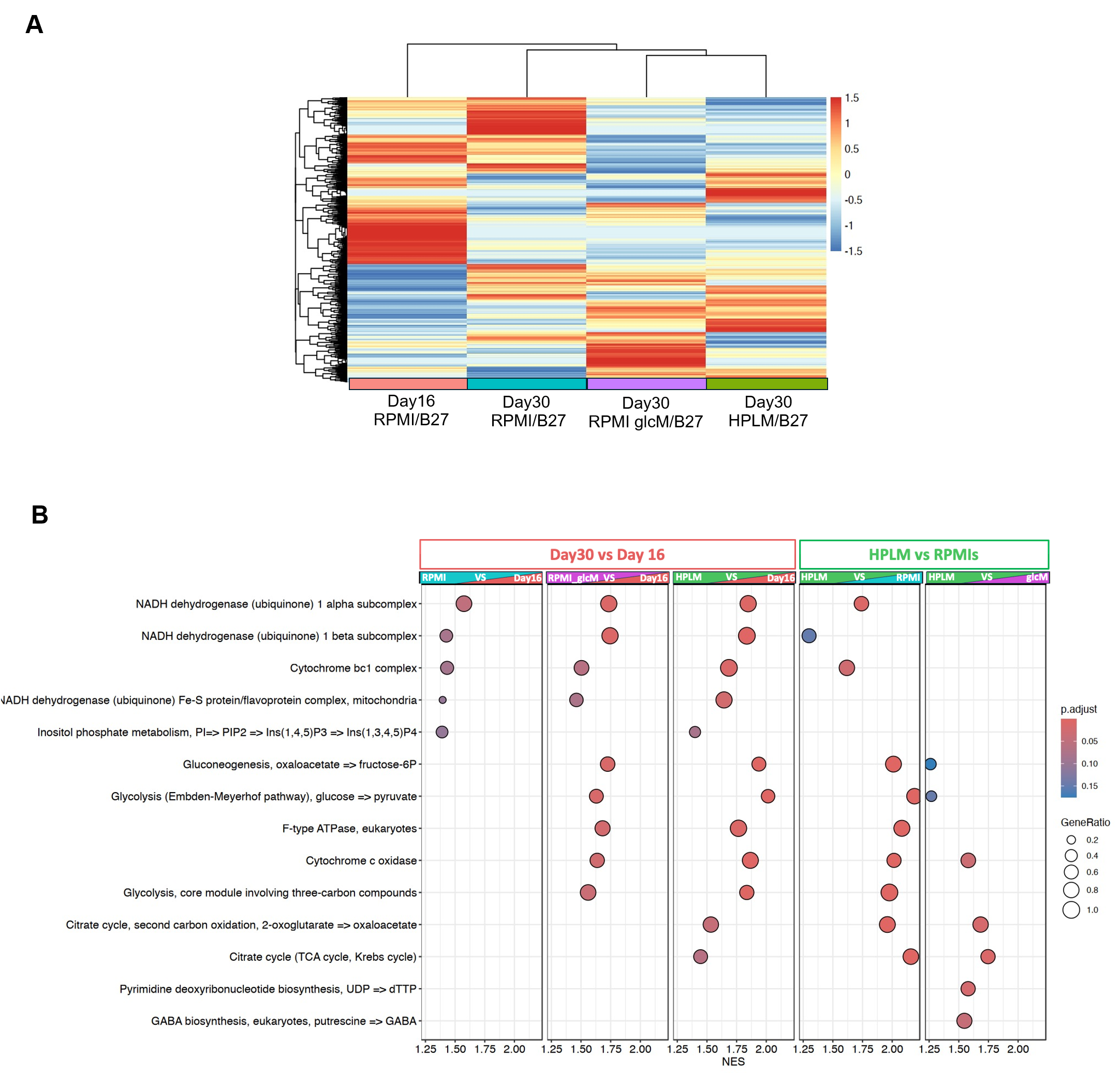
**

**Figure S2. Transcriptomic profiling of hPSC-CMs cultured in different media.** (A) Heatmap clustering of the top 1,000 genes with highest variance in Day 16 hPSC-CMs cultured in RPMI/B7, and Day 30 hPSC-CMs cultured in standard RPMI/B27, RPMI_glcM/B27, and HPLM/B27. (B) Dot plots of top upregulated metabolic KEGG pathways identified by Gene Set Enrichment Analysis (GSEA) across five experimental comparisons: (1) Day 30 RPMI/B27 vs. Day 16, (2) Day 30 RPMI/B27_glcM vs. Day 16, (3) Day 30 HPLM/B27 vs. Day 16, (4) Day 30 HPLM/B27 vs. Day 30 RPMI/B27, and (5) Day 30 HPLM/B27 vs. Day 30 RPMI/B27_glcM. Dot size reflects the number of genes associated with a pathway; color indicates adjusted p-value.


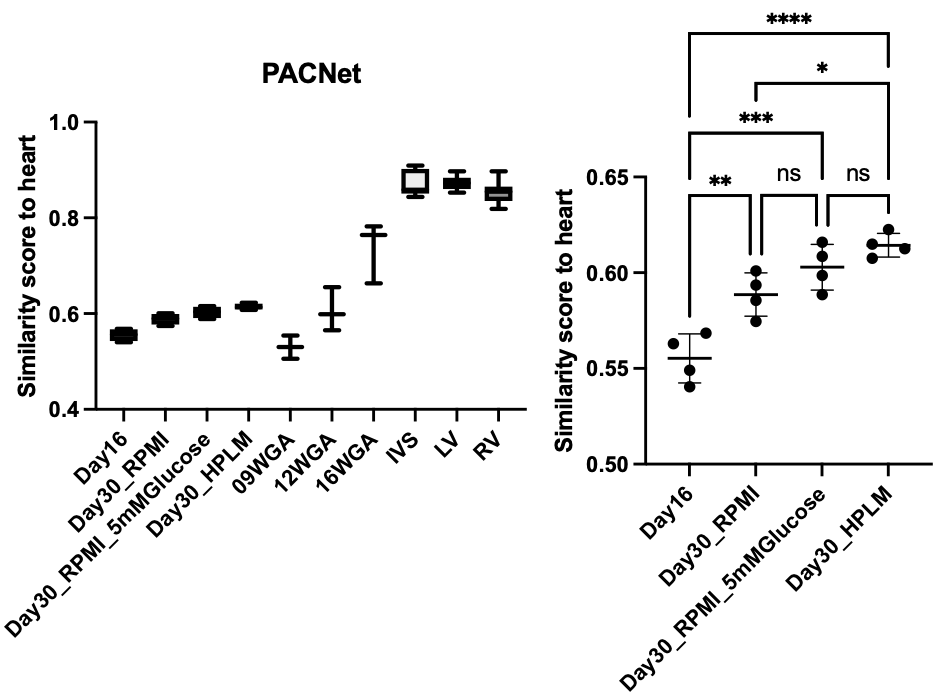


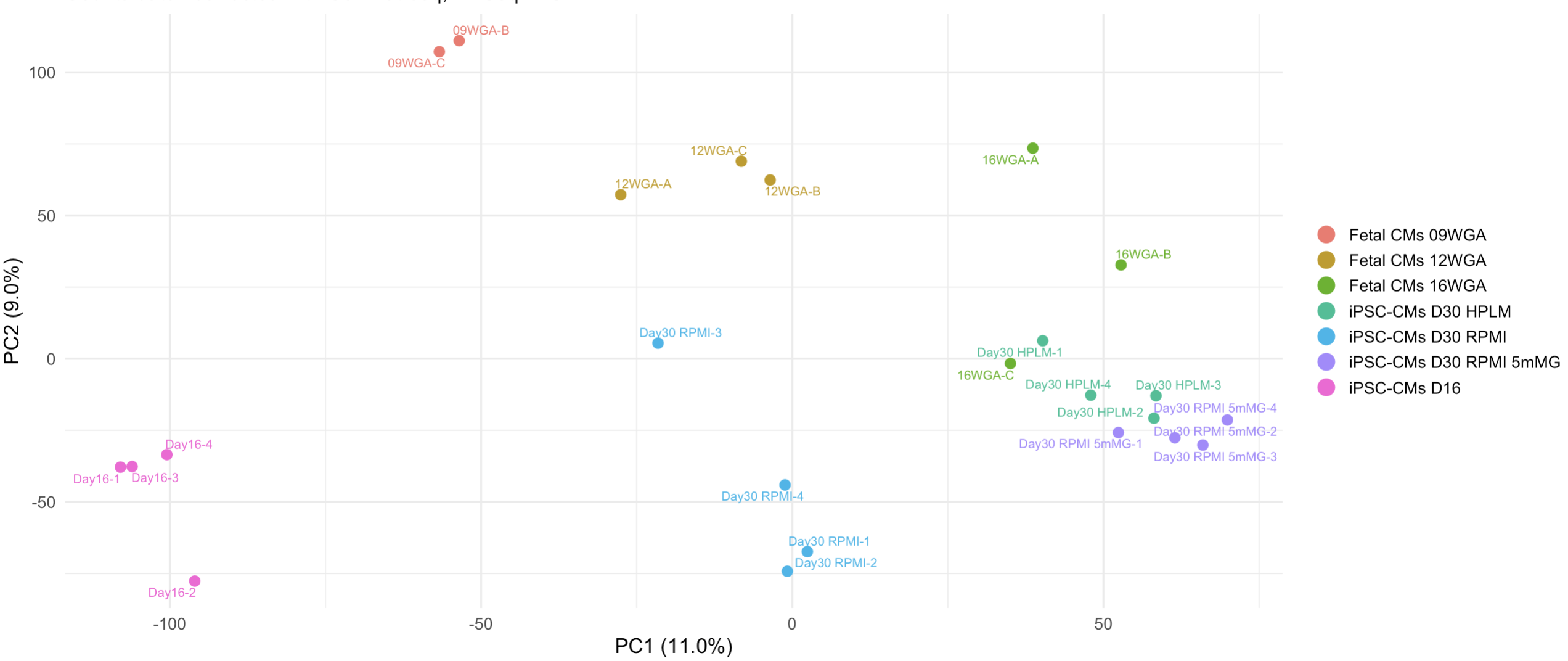


A

B

C

**Figure S3. Transcriptomic benchmarking of hPSC-CMs by media.**

(A) PACNet^4^ classification scores for hPSC-CMs across media (Day 16 RPMI/B27; Day 30 RPMI/B27; Day 30 RPMI_glcM/B27; Day 30 HPLM/B27) benchmarked against in vivo references: fetal CMs^5^ (9,12,16 weeks of gestation) and adult CMs^6^ (IVS: interventricular septum, LV: Left ventricular, RV:Right ventricular). (B) Zoom-in of PACNet scores for the in vitro groups in (A). Data are mean ± SD, statistical significance is determined by one-way ANOVA with Tukey’s post hoc test. *p<0.05, **p<0.01, ***p<0.001, ****p<0.0001 (C) Principal component analysis (PCA) of bulk RNA-seq profiles for hPSC-CMs (same media/timepoints) and fetal CMs (GW9, GW12, GW16). Raw gene-level counts were batch-corrected with ComBat-seq^7^ before the PCA. Each dot is one bulk RNA-seq sample.


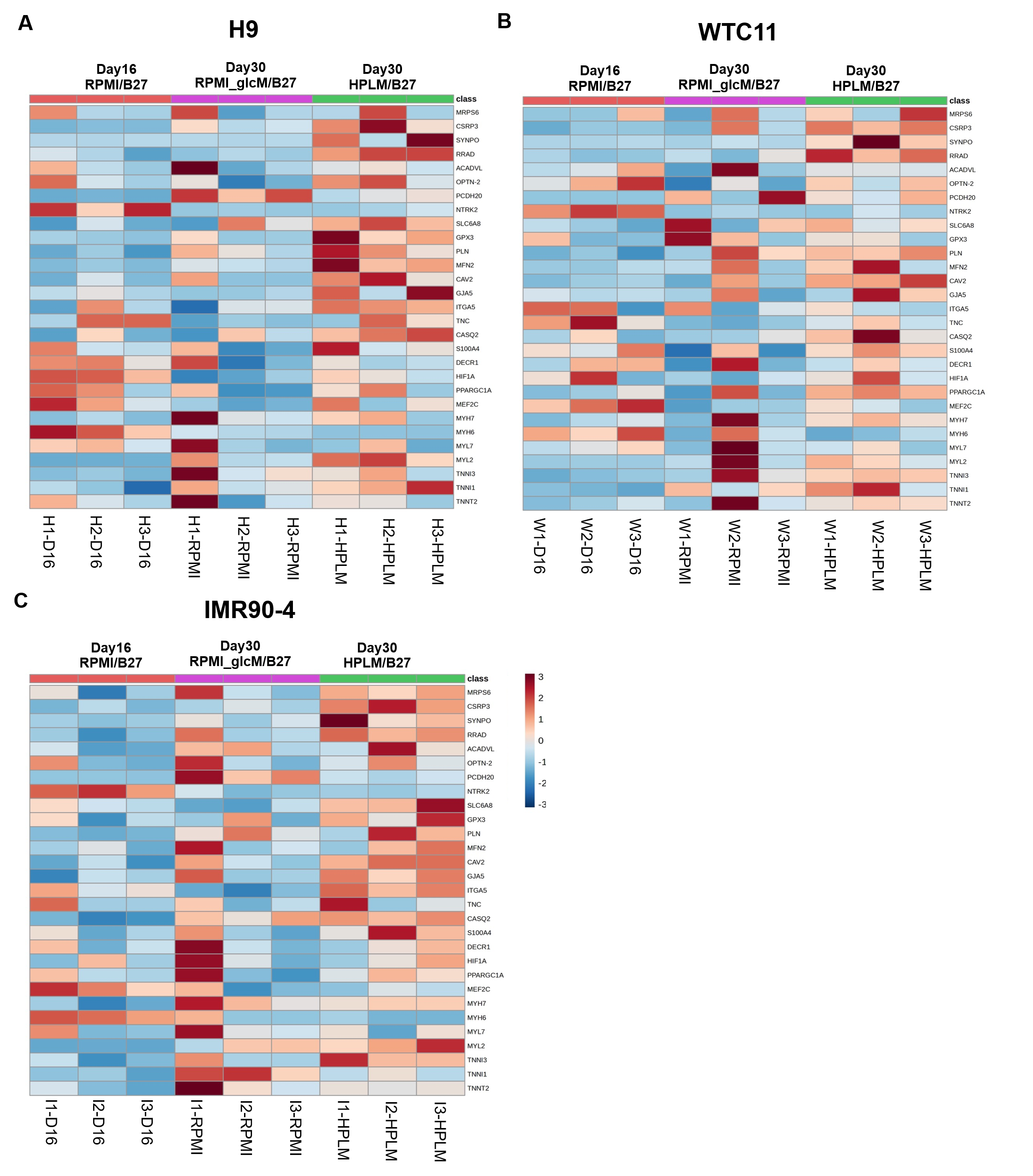


**Figure S4. qPCR validation of differentially expressed genes identified by RNA-seq across media treatments within each cell line.**

Separated Heatmap for Figure 2C of qPCR validation of differentially expressed genes identified by RNA-seq across media treatment groups: Day 16 hPSC-CMs cultured in RPMI/B27, and Day 30 hPSC-CMs cultured in RPMI_glcM/B27, and HPLM/B27. Columns (labeled H for H9, I for IMR90‐4, or W for WTC‐11) represent independent differentiations for each cell line (A)H9, (B) WTC11, (C)IMR90-4. Rows represent individual genes with the color scale indicating the Z‐score of the qPCR results (ΔCq).


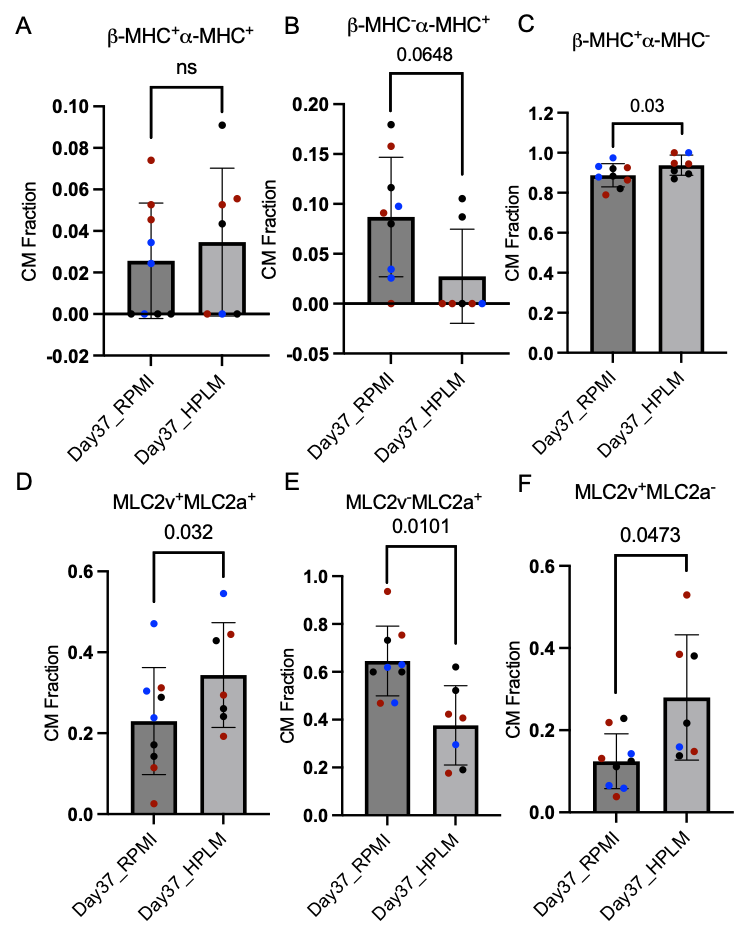


**Figure S5. Quantification of MHC and MLC isoforms immunostaining in Day 37 hPSC-CMs cultured in HPLM/B27 or RPMI_glcM/B27**

(A-C) Quantification of β-MHC and α-MHC expression in Day 37 hPSC-CMs cultured in HPLM/B27 or RPMI_glcM/B27. Blinded immunostaining images were visually analyzed to determine the fraction of cells expressing (A) both β-MHC and α-MHC (double-positive), (B) α-MHC only (single-positive), and (C) β-MHC only (single-positive). For each condition, two random fields per image were counted (≥20 cells per image), with each image derived from an independent differentiation. Data are pooled from three hPSC lines (H9, IMR90-4, WTC-11) across independent experiments. Sample sizes: RPMI_glcM/B27 (n=9 images), HPLM/B27 (n=7 images). Each dot represents one image from one independent differentiation.(D-F) Quantification of MLC2v and MLC2a expression in Day 37 hPSC-CMs cultured in HPLM/B27 or RPMI_glcM/B27. Blinded immunostaining images were visually analyzed to determine the fraction of cells expressing (D) both MLC2v and MLC2a only (double-positive), (E) MLC2a only (single-positive), or (F) MLC2v only (single-positive). For each condition, two random fields per image were counted (≥20 cells per image), with each image derived from an independent differentiation. Data are pooled from three hPSC lines (H9, IMR90-4, WTC-11) across independent experiments. Sample sizes: RPMI_glcM/B27 (n=9 images), HPLM/B27 (n=7 images). Each dot represents one image from one independent differentiation. Data are mean ± SD. p-values from a two-factor linear mixed-effects model (media, cell line) with repeated measures testing for a main media effect. Per–cell line Šídák's multiple comparisons test is reported in Table S4.


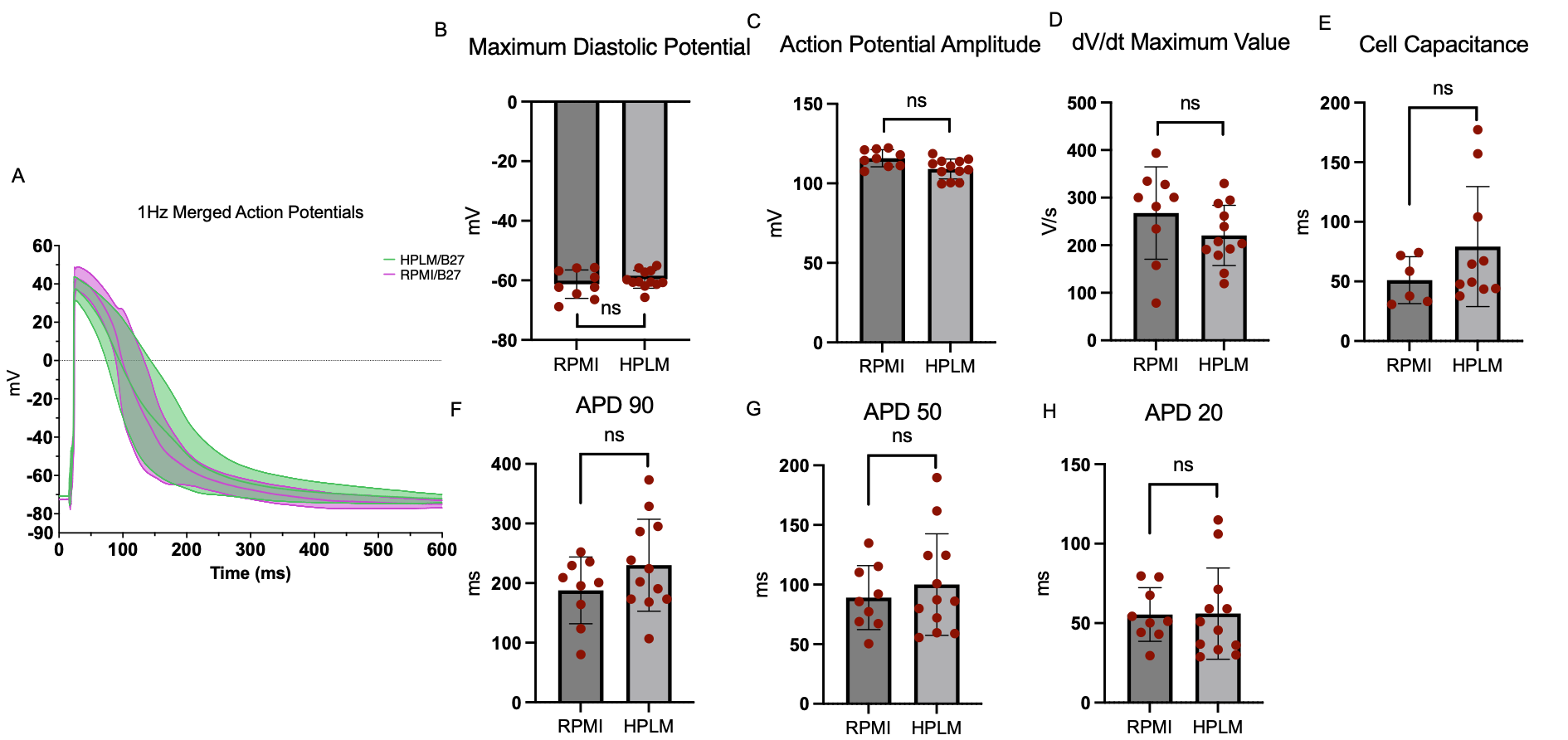


**Figure S6. Patch-clamp action potential properties of Day 30 hPSC-CMs (IMR90-4) cultured in RPMI_glcM/B27 vs HPLM/B27.**

(A) Average action potential was obtained by merging APs recorded under 1 Hz stimulation at 37 °C. Curves show mean ± SD for each medium. (n=8 cells for RPMI_glcM/B27, n=11 cells for HPLM/B27). (B–H) Single-cell AP metrics, including (B) Maximum Diastolic Potential (MDP) values are reported without correction for liquid junction potential (–11 mV), (C) Action potential Amplitude, (D) dV/dt Maximum Value, (E) Cell Capacitance, and action potential duration at (F) 90% (APD90) and (G) 50% (APD50) (H) 20% (APD20) repolarization. Each dot = one cell. mean ± SD with n=8 cells for RPMI_glcM/B27, and n=11 cells for HPLM/B27.


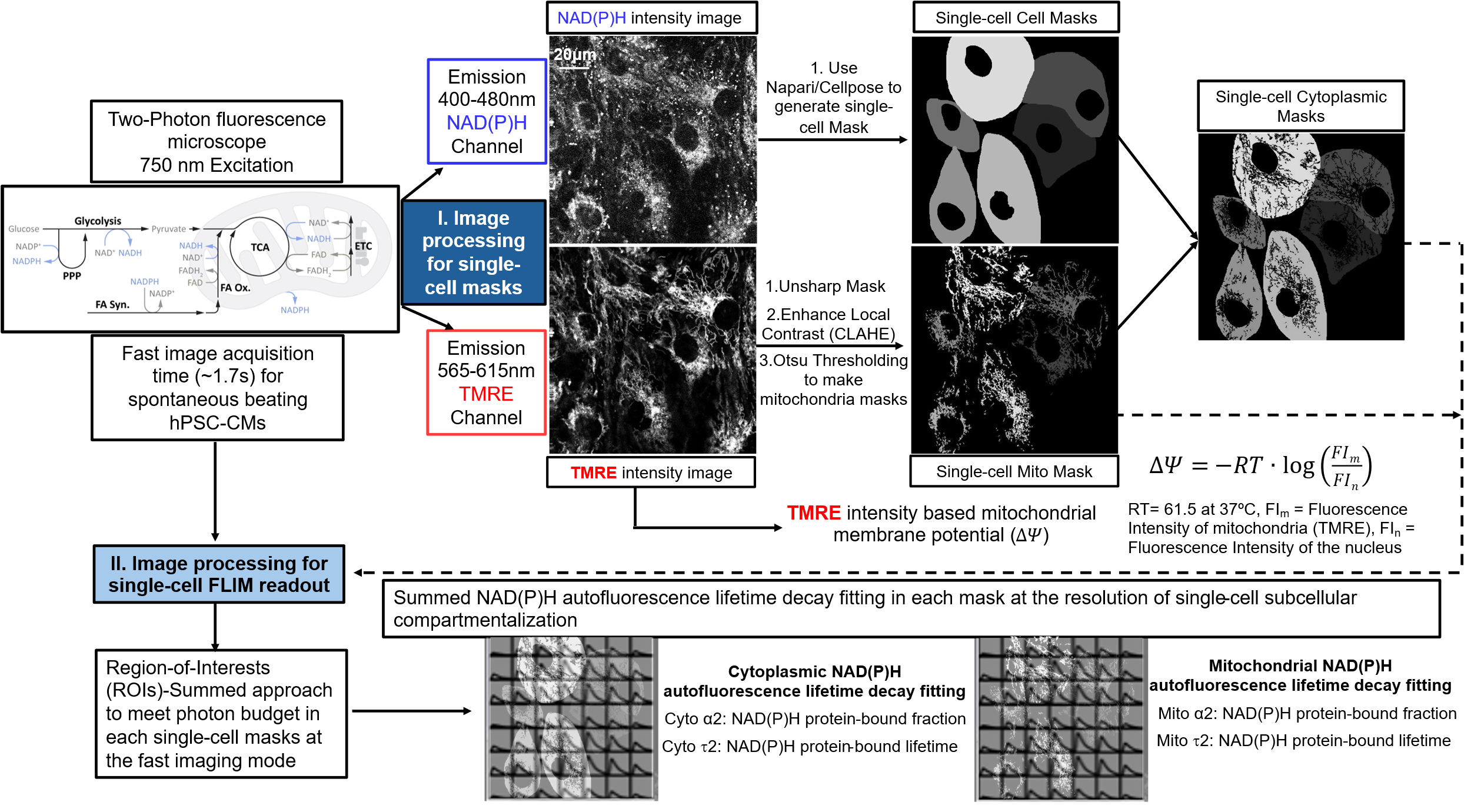


**Figure S7. Workflow of FLIM-based NAD(P)H metabolic analysis in hPSC-CMs.**

(A) Single-cell segmentation and mitochondrial membrane potential (∆Ψ) quantification. Image processing workflow for generating subcellular masks (cell, mitochondria, cytoplasm) and measuring single-cell mitochondrial membrane potential (∆Ψ) by TMRE (tetramethylrhodamine, ethyl ester). TIF-formatted NAD(P)H intensity images were imported into Napari^8^ and Cellpose^9^ to generate single-cell whole-cell and nuclear masks. Mitochondrial segmentation was performed in ImageJ from TMRE intensity images using a custom pipeline adapted from established morphological analysis methods^10^. Mitochondrial membrane potential was quantified based on TMRE intensity in mitochondrial and normalized by nuclear intensity by equation $\Delta\Psi=$ $-RT\cdot\log\left( \frac{FIm}{FIn} \right)$ followed by previous protocol ^11^. (B) NAD(P)H fluorescence lifetime analysis. Region-of-interest (ROI)-summed approach for quantifying NAD(P)H fluorescence lifetime parameters (τ2, α2) in cytoplasmic and mitochondrial compartments. To ensure sufficient photon counts for reliable decay fitting, the ROI-Summing method was implemented as detailed in prior methodology^12^. NAD(P)H fluorescence lifetime data were processed using SPCImage software, with two-component exponential decay models (
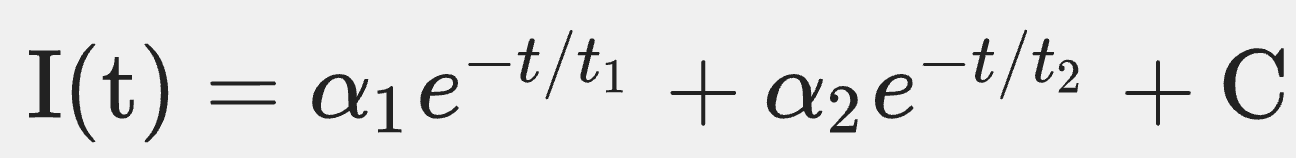
) applied as described previously ^13^. Single-cell NAD(P)H lifetime parameters (mitochondrial and cytoplasmic compartments) were extracted from SPCImage-generated ASC files using Python-based parsing and compiled into structured datasets via Excel.


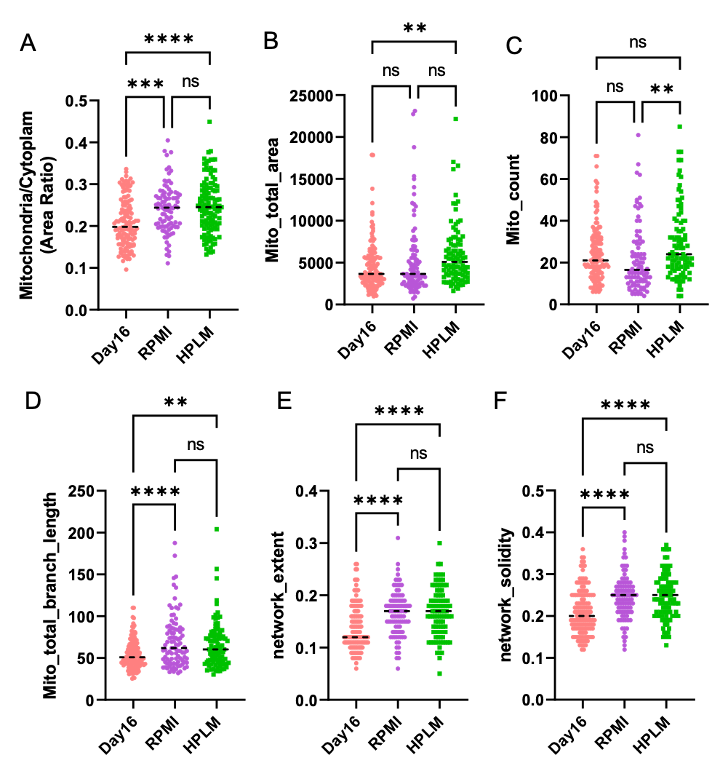


**Figure S8. Single-cell mitochondrial morphology of hPSC-CMs across culture conditions.**

(A–H) Single-cell mitochondrial morphometrics analyses^14^ including (A) Mitochondria/Cytoplasm (2D Area Ratio) (B) Total mitochondrial area (C) Total Mitochondrial count (D) Total mitochondrial branch length (E) Averaged mitochondrial extent (The ratio of mitochondrion area to the area of its bounding box) (F) Averaged mitochondrial solidity (The ratio of mitochondrion area to the area of its surrounding convex hull). Mitochondrial masks were generated from the mitochondrial channel as described in Figure S7. hPSC-CMs in Day 16 hPSC-CMs cultured in RPMI/B27, and Day 30 hPSC-CMs cultured in RPMI_glcM/B27 or HPLM/B27. Data include 90 to 130 single cells for three independent differentiation batches of H9, IMR90-4, and WTC11 hPSCs. Data are mean ± SD, statistical significance is determined by one-way ANOVA with Tukey’s post hoc test. *p<0.05, **p<0.01, ***p<0.001, ****p<0.0001

**Supporting Video Legends**

**Video S1**

Representative video of the contractility of Day 30 hPSC-CMs cultured from days 16 to 30 in RPMI/B27 containing 5 mM glucose.

**Video S2**

Representative video of the contractility of the Day 30 hPSC-CMs cultured from days 16 to 30 in HPLM/B27.

**Video S3**

Representative video of calcium transient of the Day 30 hPSC-CMs cultured from days 16 to 30 in RPMI/B27 containing 5 mM glucose.

**Video S4**

Representative video of calcium transient of the Day 30 hPSC-CMs cultured from days 16 to 30 in HPLM/B27.

**References**

1. Rossiter NJ, Huggler KS, Adelmann CH, et al. CRISPR screens in physiologic medium reveal conditionally essential genes in human cells. *Cell Metab*. 2021;33(6):1248-1263.e9. doi:10.1016/J.CMET.2021.02.005/ATTACHMENT/BBF807B9-0F90-4151-B2AD-E1654CBE90A8/MMC8.PDF

2. Cantor JR, Abu-Remaileh M, Kanarek N, et al. Physiologic Medium Rewires Cellular Metabolism and Reveals Uric Acid as an Endogenous Inhibitor of UMP Synthase. *Cell*. 2017;169(2):258-272.e17. doi:10.1016/J.CELL.2017.03.023/ATTACHMENT/2DBD023D-FB4C-485B-AC88-4EA1EA829FD2/MMC3.XLSX

3. Flickinger KM, Wilson KM, Rossiter NJ, et al. Conditional lethality profiling reveals anticancer mechanisms of action and drug-nutrient interactions. *Sci Adv*. 2024;10(40):3591. doi:10.1126/SCIADV.ADQ3591

4. Lo EKW, Velazquez JJ, Peng D, Kwon C, Ebrahimkhani MR, Cahan P. Platform-agnostic CellNet enables cross-study analysis of cell fate engineering protocols. *Stem Cell Reports*. 2023;18(8):1721-1742. doi:10.1016/J.STEMCR.2023.06.008/ASSET/AE971328-4314-4B0D-889B-26C18213A28D/MAIN.ASSETS/FX1_LRG.JPG

5. Pervolaraki E, Dachtler J, Anderson RA, Holden A V. The developmental transcriptome of the human heart. *Scientific Reports 2018 8:1*. 2018;8(1):1-9. doi:10.1038/s41598-018-33837-6

6. Johnson EK, Matkovich SJ, Nerbonne JM. Regional Differences in mRNA and lncRNA Expression Profiles in Non-Failing Human Atria and Ventricles. *Scientific Reports 2018 8:1*. 2018;8(1):1-13. doi:10.1038/s41598-018-32154-2

7. Zhang Y, Parmigiani G, Johnson WE. ComBat-seq: batch effect adjustment for RNA-seq count data. *NAR Genom Bioinform*. 2020;2(3). doi:10.1093/NARGAB/LQAA078

8. Sofroniew N, Lambert T, Bokota G, et al. napari: a multi-dimensional image viewer for Python. doi:10.5281/ZENODO.15029515

9. Stringer C, Wang T, Michaelos M, Pachitariu M. Cellpose: a generalist algorithm for cellular segmentation. *Nature Methods 2020 18:1*. 2020;18(1):100-106. doi:10.1038/s41592-020-01018-x

10. Valente AJ, Maddalena LA, Robb EL, Moradi F, Stuart JA. A simple ImageJ macro tool for analyzing mitochondrial network morphology in mammalian cell culture. *Acta Histochem*. 2017;119(3):315-326. doi:10.1016/J.ACTHIS.2017.03.001

11. Gillette AA, DeStefanis RA, Pritzl SL, Deming DA, Skala MC. Inhibition of B-cell lymphoma 2 family proteins alters optical redox ratio, mitochondrial polarization, and cell energetics independent of cell state. *https://doi.org/101117/1JBO275056505*. 2022;27(5):056505. doi:10.1117/1.JBO.27.5.056505

12. Samimi K, Desa DE, Pham DL, Skala MC. ROI-summed analysis for improved fluorescence lifetime fitting accuracy and precision. Periasamy A, So PT, König K, eds. *Multiphoton Microscopy in the Biomedical Sciences XXV*. Published online March 20, 2025:48. doi:10.1117/12.3043430

13. Qian T, Heaster TM, Houghtaling AR, Sun K, Samimi K, Skala MC. Label-free imaging for quality control of cardiomyocyte differentiation. *Nature Communications 2021 12:1*. 2021;12(1):1-11. doi:10.1038/s41467-021-24868-1

14. Rohani A, Kashatus JA, Sessions DT, Sharmin S, Kashatus DF. Mito Hacker: a set of tools to enable high-throughput analysis of mitochondrial network morphology. *Scientific Reports 2020 10:1*. 2020;10(1):1-15. doi:10.1038/s41598-020-75899-5
